# Screening of Genetic Factor in the Interaction Between Periodontitis and Metabolic Traits Using Candidate Gene Association Study (CGAS)

**DOI:** 10.1007/s10528-018-9899-9

**Published:** 2018-12-13

**Authors:** Kyung-Hui Moon

**Affiliations:** Department of Dental Hygiene, Jinju Health College, Uibyeong-ro 51, Jinju, Korea

**Keywords:** Korean, Periodontitis, *A2BP1*, *TENM2*, *LDLRAD4*, Metabolic traits

## Abstract

Periodontitis has been reported to relate to metabolic syndrome traits such as obesity, blood pressure, and so on. However, the relation between periodontitis and metabolic syndrome remains unclear. The present study aimed to confirm common genetic factors between periodontitis and metabolic traits using Candidate gene association study (CGAS) in the Korean population. Based on the analysis of CGAS, this study performed linear regression analyses to examine the single-nucleotide polymorphisms (SNPs) between periodontitis and metabolic syndrome traits. Among the analyzed SNPs, 2649 SNPs in five genes (*TENM2*, *LDLRAD4*, *SLC9C2*, *MFSD1*, and *A2BP1*) showed a statistical significance at *p* < 0.05. Interestingly, *A2BP1* and *TENM2* were related to obesity. Also, elevated levels of *LDLRAD4*, *SLC9C2,* and *MFSD1* were observed in the patients with high blood pressure. Taken together, the present study suggests that some of the SNPs are related to periodontitis. Therefore, if any *of TENM2*, *A2BP1*, *LDLRAD4*, *SLC9C2,* and *MFSD1* is detected in the patients with periodontitis, obesity and blood pressure have to be treated simultaneously.

**Electronic supplementary material:**

The online version of this article (10.1007/s10528-018-9899-9) contains supplementary material, which is available to authorized users.

## Introduction

Periodontitis is a chronic inflammatory disease by bacterial infection of the tissues supporting the teeth (Haffajee and Socransky 1994; Page and Kornman 1997). It was reported that the development of periodontitis is an irreversible process (Nelson-Filho et al. 2018). Unfortunately, it is a major cause of tooth loss in adults. Periodontitis in the Korean population accounted for amount to 10.2% to 55.7% depending on age (Kim et al. 2014).

Interestingly, major cause of periodontitis was various diseases, such as dyslipidemia, glucose intolerance, hypertension, and a low-grade systemic inflammatory state (Winning and Linden 2017; Lamster and Pagan 2017), as well as with systemic diseases and conditions termed metabolic traits such as cardiovascular disease, diabetes, and obesity (Hong et al. 2015). Furthermore, a number of studies have shown that periodontitis resulted from periodontal microorganisms and smoking (Socransky et al. 1998; Socransky and Haffajee 2005; Gelskey 1999). Recent a study using cross-sectional and longitudinal designs demonstrated an association between periodontitis and metabolic traits (Nibali et al. 2013). However, there are contradictory reports showing no association between periodontitis and metabolic traits (Nibali et al. 2013). This might be due to age, gender, socioeconomic status genetic factor, and lifestyle (Genco and Borgnakke 2013). Nevertheless, the genes inducing periodontitis have already been known as genetic risk markers of multifactorial diseases (Sanders et al. 2017; Hong et al. 2015).

Interestingly, a genetic study on CGAS of the Korean periodontitis has focused upon ten periodontitis genes (*TENM2*, *LDLRAD4*, *SLC9C2*, *RASGRP4*, *MFSD1*, *IL4*, *NMUR2*, *GPR141*, *GLK*, and *A2BP1*) identified as the potential candidate genes with genetic risk factors (see Table 1) (Hong et al. 2015).

**Table 1:** Reported genetic risk factors in Korean Periodontitis

SNP	Gene	Effect of size	*p* value	Ref 1	Ref 2	Ref 3
rs4242220	TENM2	0.53	2.84 × 10^−6^	Hong et al. (2015)		
rs12969041	LDLRAD4	2.86	2.79 × 10^−7^	Hong et al. (2015)		
rs2027756	LDLRAD4	2.86	2.79 × 10^−6^	Hong et al. (2015)		
rs16846206	SLC9C2	2.02	7.66 × 10^−5^	Hong et al. (2015)		
rs892055	RASGRP4	0.49	1.23 × 10^−4^	Hong et al. (2015)		
rs1346834	MFSD1	0.71	0.007	Hong et al. (2015)	Teumer et al. (2013)	
rs2243250	IL4	0.65	0.004	Hong et al. (2015)	Laine et al. (2012)	Divaris et al. (2013)
rs2070874	IL4	0.66	0.006	Hong et al. (2015)	Laine et al. (2012)	Divaris et al. (2013)
rs294958	NMUR2	1.29	0.034	Hong et al. (2015)	Teumer et al. (2013)	
rs2392510	GPR141	1.48	9.48 × 10^−4^	Hong et al. (2015)	Shimizu et al. (2015)	
rs2243407	BLK	0.73	0.01	Hong et al. (2015)	Teumer et al. (2013)	
rs11866781	A2BP1	1.32	0.045	Hong et al. (2015)	Teumer et al. (2013)	

Because periodontitis triggered metabolic syndrome traits, the roles of these genes in periodontitis should be confirmed. Nevertheless, the functions of these genes have not been clearly understood so far.

To clearly verify the relation between periodontitis and metabolic traits, single-nucleotide polymorphisms (SNPs) were investigated after the screening of the candidate genes. However, considering that gene was repetitively replicated, the SNP study did not provide convincing evidence for the presence of risk alleles.

Candidate gene association study (CGAS) is a bias-free approach for the identification of risk genes. Recently, a study analyzed four genetic associations in chronic European and Japanese populations (Giacomini et al. 2017), and then the most harmful risk alleles were identified by confirming more than 30 promising loci and candidate genes on periodontal health and diseases. Based on these results, it is necessary to validate genetic association in other countries. Hence, the aim of the present study is to confirm genetic association reported between periodontitis and metabolic traits in a Korean cohort.

## Materials and Methods

### Study Subjects

The participants of this study were recruited from the Korean Genome and Epidemiology study (KoGES) project, a national project to perform genome epidemiology studies in cohorts of Korean dwellers and immigrants/emigrants (Kim et al. 2017). Among the KoGES cohorts, a public genetic information dataset was established by the Korean Association Resource Consortium (KARE) based on the Ansan–Anseong cohort. This cohort is biennially followed up in the ongoing KoGES project (Karns et al. 2012). The KARE dataset consists of individual SNP chip genotypes and the epidemiological/clinical phenotypes for study of the genetic components of the Korean public health. Written informed consent was obtained from all the participants included at the KoGES. The obtained KARE dataset was analyzed based on the standard (inclusion/exclusion) criteria (Cho et al. 2009). In short, subjects with the genotyping accuracy below 98% and high extent of missing genotype call rates (≥ 5%), as well as high heterozygosity (> 30%) or inconsistency in gender-based data were excluded from subsequent analysis. Furthermore, this study excluded individuals with tumor, as well as those individuals whose estimated identity-by-state values were high (> 0.80). Based on these factors of criteria, a total of 8842 participants were identified as eligible for inclusion and screened for the purpose of our study.

### Study Phenotypes and Covariates

A study using the CGAS measured the phenotypes and covariates (Jeong et al. 2014). First, the current study investigated the general demographic data based on resident areas (Anseong or Ansan), gender, and age as the covariates. In the next step, height and body weight were analyzed to calculate body mass index (BMI). Subsequently, the waist circumference (WC), systolic and diastolic blood pressures (SBP and DBP), fasting plasma glucose levels (GLU0), high-density lipoprotein (HDL) cholesterol, and triglyceride (TG) were measured for the genetic association study.

### Study Genotypes

A previous study used an approach for genotyping of the cohort population for the KARE study (Kim et al. 2017). Moreover, those researchers isolated most of the DNA samples from the peripheral blood of the participants and genotyped them using Affymetrix Gene-wide Human SNP array 5.0 (Affymetrix, Inc., Santa Clara, CA, USA). The current study postulated that genetic association can be discovered using the quality-control steps X of the genotypes. To test this assumption, this study applied the Bayesian Robust Linear Modeling with the Mahalanobis Distance genotyping algorithm to determine the call rate of the genotyping. Consequently, 352,227 SNPs had a missing genotype call rate below 0.1, a minor allele frequency greater than 0.01, and no deviation from Hardy–Weinberg equilibrium (*p* > 1 × 10^−6^). In addition, CGAS reported no population stratification between the Anseong and Ansan cohorts (Cho et al. 2009).

### Statistical Analyses

Linear regression analysis was performed for the current genetic association study on the basis of residential area, gender, and age. Afterward, statistical analyses were performed using PLINK (version 1.07) (Purcell et al. 2007). This study determined the significant associations using an unadjusted *p* value (< 0.05).

## Results

### Genetic Association Study Between Periodontitis Genes and Metabolic Traits

To confirm genetic association between periodontitis genes and metabolic traits in the Korean population, this study investigated ten periodontitis genes known as genetic risk factors and as clinical characteristics of metabolic traits (see Tables 1 and 2) (Hong et al. 2015). Based on these data, gene regions were determined (see Table 3). A total 10,268 SNPs of ten targeted periodontitis genes were analyzed among 2649 SNPs in five genes (*TENM2*, *LDLRAD4*, *SLC9C2*, *MFSD1*, and *A2BP1*), and their statistical differences (*p* < 0.05) are listed (Supplementary Table 1). In terms of the association with BMI, waist circumference (WAIST), and fasting glucose (FG), 2592 of 2,6S9 SNPs were located in A2BP1 and showed a statistical significance (*p* < 0.01) with respect to the obesity-phenotypes. Also, the presented study found that one SNP (rs138566395) showed statistical significance at *p* < 0.01 (see Table 4A) in the relationship between triglycerides (TG) and high-density lipoprotein (HDL) cholesterol. Interestingly, these SNPs were related to BMI (*β* ± SE = − 0.23 ± 0.06, *p* = 0.00022) and WAIST and HDL (see Tables 4A and 4C).

**Table 2:** Characteristics of clinical metabolic traits

List	Cohort	The analysis model
No. of individuals	8842	
Resident area (Anseong %)	47.5	Covariates
Gender (male %)	47.3	Covariates
Age (years old)	52.2 ± 8.9	Covariates
Height (cm)	160.0 ± 8.7	
Body mass index (kg/m^2^)	24.6 ± 3.1	Target 1
Fasting glucose (mg/dL)	87.7 ± 21.9	Target 2
DBP (mmHg)	80.3 ± 11.5	Target 3
SBP (mmHg)	121.7 ± 18.6	Target 4
Waist circumference (cm)	82.7 ± 8.8	Target 5
HDL cholesterol (mg/dL)	44.7 ± 10.1	Target 6
Triglyceride (mg/dL)	162.9 ± 105.7	Target 7

**Table 3:** Targeted gene regions

Periodontitis genes	Chr	Gene regions		Analysis region (gene region ± 5 kbp)		
		Start	End	Start	End	SNPs
TENM2	5	166711843	167691162	166706843	167696162	103
LDLRAD4	18	13218729	13652753	13213729	13657753	77
SLC9C2	1	173469604	173572233	173464604	173577233	245
RASGRP4	19	38899698	38916945	38894698	38921945	95
MFSD1	3	158519715	158547508	158514715	158552508	105
IL4	5	132009678	132018370	132004678	132023370	48
NMUR2	5	151771102	151784840	151766102	151789840	115
GPR141	7	37723378	37783423	37718378	37788423	198
BLK	8	11351501	11422113	11346501	11427113	176
A2BP1 (RBFOX1)	16	6069132	7763340	6064132	7768340	9106

**Table 4:** Adjusted association of genomewide association study between periodontitis genes and metabolic traits

(A) Genomewide association with body mass index (BMI), waist circumference (WAIST), and fasting glucose (FG)														
	CHR	SNP	BP	A1	NMISS	BMI	SE	P	WAIST	SE	P	FG	SE	P
A2BP1	16	rs138566395	7323241	C	8836	− 0.23	0.06	2.20E−04	− 0.61	0.17	3.50E−04	− 0.69	0.47	1.40E−01
TENM2	5	rs1529682	167486071	G	8831	− 0.33	0.15	2.50E−02	− 1.19	0.4	2.90E−03	1.25	1.11	2.60E−01
TENM2	5	rs1477284	167217586	T	8835	− 0.15	0.06	9.90E−03	− 0.33	0.16	3.90E−02	0.1	0.44	8.20E−01
TENM2	5	rs3733986	167585972	T	8834	− 0.05	0.05	3.40E−01	− 0.08	0.13	5.60E−01	− 1.24	0.37	8.30E−04

(B) Genomewide association with diastolic blood pressure (DBP), and systolic blood pressure (SBP)											
	CHR	SNP	BP	A1	NMISS	DBP	SE	P	SBP	SE	P
LDLRAD4	18	rs3132835	13496046	C	8836	0.53	0.19	5.10E−03	0.81	0.29	4.60E−03
MFSD1	3	rs2061617	158523819	G	8836	2.33	0.73	1.50E−03	2.47	1.1	2.50E−02
SLC9C2	1	rs190870441	173568389	G	8836	− 1.45	0.68	3.30E−02	− 3.03	1.02	3.00E−03

(C) Genomewide association with triglycerides (TG) and high-density lipoprotein (HDL) cholesterol											
	CHR	SNP	BP	A1	NMISS	TG	SE	P	HDL	SE	P
A2BP1	16	rs138566395	7323241	C	8836	− 4.77	2.21	3.10E−02	0.64	0.24	7.10E−03
LDLRAD4	18	rs3931961	13393109	C	8830	− 4.87	1.71	4.40E−03	0.33	0.18	7.40E−02

Next, this study also showed that three SNPs (rs1529682, rs147728, and rs3733986) of *TENM2* were associated with BMI, WAIST, and FG (see Table 4A). Moreover, *LDLRAD4*, *SLC9C2*, and *MFSD1* were related to DBP and SBP in triglycerides (TG) and high-density lipoprotein cholesterol (see Table 4B).

## Discussion

The present study discovered that five genes (*A2BP1*, *TENM2*, *LDLRAD4*, *SLC9C2*, and *MFSD1*) were associated with metabolic traits such as obesity and high blood pressures. Also, these genes were strongly associated with BMI (*p* = 2.2 × 10^−4^). However, when either Bonferroni correction or false discovery rate correction was applied, no statistical significance was observed. This may be the reason that metabolic traits can be determined by age, gender, socioeconomic status, genetic factor, and lifestyle. Based on these facts, the present study focused on investigating the genetic association between periodontitis genes and metabolic traits.

This study demonstrated that *A2BP1* was strongly associated with periodontitis. Therefore, this gene could be considered as a genetic risk marker. In the United States, *A2BP1* is already reported as a genetic risk marker (Purcell et al. 2007). *A2BP1* encoding Ataxin 2-binding protein 1 is known as RNA-binding fox-1 Homolog 1. Reduction of this gene in mouse hypothalamus cells led to decreases in the expressions of ATXN2, INSR, and MC4R, which have important roles in the metabolic pathways (Ma et al. 2010). Conversely, increased *A2BP1* expression was found in obesity, Also, this gene interacted with ATXN2, INSR, and MC4R, which played important roles in metabolic pathways (Ma et al. 2010). Interestingly, rs138566395 on third intron of the gene in the *A2BP1* region showed significant associations with BMI, HDL cholesterol, Triglycerides, and WAIST (see Table 4).

*TENM2* encoding teneurin-transmembrane protein 2 was strongly associated with periodontitis. In line with this finding, a recent study showed that *TENM2* was dramatically increased in the adipocyte progenitor cells. Moreover, *TENM2*-deficiency upregulated brown adipocyte marker genes (Tews et al. 2017), which indicated that *TENM2* could contribute to cell fate. *TENM2* was reported to act as a membrane-bound transcriptional regulator in the intracellular role and inhibit zic-mediated transcription by stimulating the apolipoprotein E (APOE) promoter (Bagutti et al. 2003). Induction of APOE was observed in the patients with *Porphyromonas gingivalis* (Lei et al. 2013), which indicated that periodontitis might induce metabolic syndrome via *TENM2*-activated APOE promoter. In addition, it was reported that *TENM2* was related to *subgingival Aggregatibacter actinomycetemcomitans* known as one of the periodontal pathogens (Divaris et al. 2012). Besides, this gene was important in maintaining serum uric acid concentration (Karns et al. 2012).

*LDLRAD4*, *MFSD1*, and *SLC9C2* were strongly associated with DBP and SBP. Particularly, *LDLRAD4* was observed in the patients with blood pressure (SBP and DBP). Reportedly, this gene consisted of low-density lipoprotein receptor (LDLR) class A domains which are the major cholesterol-carrying lipoproteins of plasma. Besides, LDL binds class A domain to the LDLR, and then transports it into cells (Van der Horst et al. 2009). Therefore, the *LDLRAD4* was critical in cholesterol homeostasis in mammalian cells. In addition, because the LDLR class A domain is a binding site for calcium, numerous familial hypercholesterolemia mutations of the LDL receptor could alter the calcium-coordinating residue of LDL-A domains (Yamamoto and Yamashita 1998). Therefore, a significant upregulation of *LDLRAD4* in periodontitis could disrupt intracellular homeostasis of calcium. Based on these facts, this study suggested that *LDLRAD4* expression was important for maintaining cholesterol homeostasis in mammalian cells.

In summary, the present study showed that gene triggering periodontitis was related to obesity and blood pressure using the analysis of SNPs. Specifically, if any one of *TENM2*, *A2BP1*, *LDLRAD4*, *SLC9C2*, or *MFSD1* was observed in the patients with periodontitis, obesity and blood pressure have to be treated simultaneously.

## Electronic supplementary material

Below is the link to the electronic supplementary material.

Supplementary material 1 (XLSX 624 kb)

## References

[CR1] Bagutti C, Forro G, Ferralli J, Rubin B, Chiquet-Ehrismann R (2003). The intracellular domain of teneurin-2 has a nuclear function and represses zic-1-mediated transcription. J Cell Sci.

[CR2] Cho YS, Go MJ, Kim YJ, Heo JY, Oh JH, Ban HJ, Yoon D, Lee MH, Kim DJ, Park M, Cha SH, Kim JW, Han BG, Min H, Ahn Y, Park MS, Han HR, Jang HY, Cho EY, Lee JE, Cho NH, Shin C, Park T, Park JW, Lee JK, Cardon L, Clarke G, McCarthy MI, Lee JY, Lee JK, Oh B, Kim HL (2009). A large-scale gene wide association study of Asian populations uncovers genetic factors influencing eight quantitative traits. Nat Genet.

[CR3] Divaris K, Monda KL, North KE, Olshan AF, Lange EM, Moss K, Barros SP, Beck JD, Offenbacher S (2012). Gene wide association study of periodontal pathogen colonization. J Dent Res.

[CR30] Divaris K, Monda KL, North KE, Olshan AF, Reynolds LM, Hsueh WC, Lange EM, Moss K, Barros SP, Weyant RJ, Liu Y, Newman AB, Beck JD, Offenbacher S (2013). Exploring the genetic basis of chronic periodontitis: a genome-wide association study. Hum Mol Genet.

[CR4] Gelskey SC (1999). Cigarette smoking and periodontitis: methodology to assess the strength of evidence in support of a causal association. Commun Dent Oral Epidemiol.

[CR5] Genco RJ, Borgnakke WS (2013). Risk factors for periodontal disease. Periodontol 2000.

[CR6] Giacomini KM, Yee SW, Mushiroda T, Weinshilboum RM, Ratain MJ, Kubo M (2017). Gene wide association studies of drug response and toxicity: an opportunity for genome medicine. Nat Rev Drug Discov..

[CR70] Haffajee AD, Socransky SS (1994). Microbial etiological agents of destructive periodontal diseases. Periodontol 2000.

[CR7] Hong KW, Shin MS, Ahn YB, Lee HJ, Kim HD (2015). Genomewide association study on chronic periodontitis in Korean population: results from the Yangpyeong health cohort. J Clin Periodontol.

[CR8] Jeong SW, Chung M, Park SJ, Cho SB, Hong KW (2014). Gene-wide association study of metabolic syndrome in Koreans. Genomics Inform..

[CR9] Karns R, Zhang G, Sun G, Rao Indugula S, Cheng H, Havas-Augustin D, Novokmet N, Rudan D, Durakovic Z, Missoni S, Chakraborty R, Rudan P, Deka R (2012). Gene-wide association of serum uric acid concentration: replication of sequence variants in an island population of the Adriatic coast of Croatia. Ann Hum Genet.

[CR10] Kim Y, Han BG, KoGES group (2017). Cohort profile: the Korean Genome and Epidemiology Study (KoGES) Consortium. Int J Epidemiol.

[CR72] Kim DW, Park JC, Rim TT, Jung UW, Kim CS, Donos N, Cha IH, Choi SH (2014). Socioeconomic disparities of periodontitis in Koreans based on the KNHANES IV. Oral Dis.

[CR11] Lamster IB, Pagan M (2017). Periodontal disease and the metabolic syndrome. Int Dent J.

[CR110] Laine ML, Crielaard W, Loos BG (2012). Genetic susceptibility to periodontitis. Periodontol 2000.

[CR12] Lei L, Li H, Yan F, Xiao Y (2013). Hyperlipidemia impaired innate immune response to periodontal pathogen *Porphyromonas gingivalis* in apolipoprotein E knockout mice. PLoS ONE.

[CR13] Ma L, Hanson RL, Traurig MT, Muller YL, Kaur BP, Perez JM, Meyre D, Fu M, Körner A, Franks PW, Kiess W, Kobes S, Knowler WC, Kovacs P, Froguel P, Shuldiner AR, Bogardus C, Baier LJ (2010). Evaluation of A2BP1 as an obesity gene. Diabetes.

[CR14] Nelson-Filho P, Ruviére DB, de Queiroz AM, de Paula-Silva FWG, Silva RABD, Lucisano MP, da Silva LAB (2018). Comparative molecular analysis of Gram-negative bacteria in primary teeth with irreversible pulpitis or periapical pathology. Pediatr Dent.

[CR15] Nibali L, Tatarakis N, Needleman I, Tu YK, D’Aiuto F, Rizzo M, Donos N (2013). Clinical review: association between metabolic syndrome and periodontitis: a systematic review and meta-analysis. J Clin Endocrinol Metab.

[CR71] Page RC, Kornman KS (1997). The pathogenesis of human periodontitis: an introduction. Periodontol 2000.

[CR16] Purcell S, Neale B, Todd-Brown K, Thomas L, Ferreira MA, Bender D, Maller J, Sklar P, de Bakker PI, Daly MJ, Sham PC (2007). PLINK: a tool set for whole-genome association and population-based linkage analyses. Am J Hum Genet.

[CR17] Sanders AE, Sofer T, Wong Q, Kerr KF, Agler C, Shaffer JR, Beck JD, Offenbacher S, Salazar CR, North KE, Marazita ML, Laurie CC, Singer RH, Cai J, Finlayson TL, Divaris K (2017). Chronic periodontitis gene wide association study in the Hispanic Community Health Study Study of Latinos. J Dent Res.

[CR201] Shimizu S, Momozawa Y, Takahashi A, Nagasawa T, Ashikawa K, Terada Y, Izumi Y, Kobayashi H, Tsuji M, Kubo M, Furuichi Y (2013). A genome-wide association study of periodontitis in a Japanese population. J Dent Res.

[CR18] Socransky SS, Haffajee AD (2005). Periodontal microbial ecology. Periodontol 2000.

[CR19] Socransky SS, Haffajee AD, Cugini MA, Smith C, Kent RL (1998). Microbial complexes in subgingival plaque. J Clin Periodontol.

[CR200] Teumer A, Ernst FD, Wiechert A, Uhr K, Nauck M, Petersmann A, Völzke H, Völker U, Homuth G (2013). Comparison of genotyping using pooled DNA samples (allelotyping) and individual genotyping using the affymetrix genome-wide human SNP array 6.0. BMC Genomics.

[CR20] Tews D, Fromme T, Keuper M, Hofmann SM, Debatin KM, Klingenspor M, Wabitsch M, Fischer-Posovszky P (2017). Teneurin-2 (TENM2) deficiency induces UCP1 expression in differentiating human fat cells. Mol Cell Endocrinol.

[CR21] Van der Horst DJ, Roosendaal SD, Rodenburg KW (2009). Circulatory lipid transport: lipoprotein assembly and function from an evolutionary perspective. Mol Cell Biochem.

[CR22] Winning L, Linden GJ (2017). Periodontitis and systemic disease: association or causality?. Curr Oral Health Rep.

[CR23] Yamamoto T, Yamashita T (1998). Low-density lipoprotein apheresis using the Liposorber system: features of the system and clinical benefits. Ther Apher.

